# The m6A methylation profiles of immune cells in type 1 diabetes mellitus

**DOI:** 10.3389/fimmu.2022.1030728

**Published:** 2022-11-15

**Authors:** Yimeng Wang, Linling Xu, Shuoming Luo, Xiaoxiao Sun, Jiaqi Li, Haipeng Pang, Jun Zhou, Yuemin Zhou, Xiajie Shi, Xia Li, Gan Huang, Zhiguo Xie, Zhiguang Zhou

**Affiliations:** National Clinical Research Center for Metabolic Diseases, Key Laboratory of Diabetes Immunology (Central South University), Ministry of Education, Department of Metabolism and Endocrinology, The Second Xiangya Hospital of Central South University, Changsha, Hunan, China

**Keywords:** autoimmune diseases, type 1 diabetes mellitus, epigenetic regulation, N6-methyladenosine, immunity

## Abstract

**Background:**

Type 1 diabetes mellitus (T1DM) is caused by immune cell-mediated β-cell dysfunction. In recent decades, N6-methyladenosine (m6A) has attracted widespread attention in the scientific research field because it plays vital roles in the pathogenesis of immunity-related diseases, including autoimmune diseases. However, neither the m6A modification profile nor the potential role it plays in T1DM pathogenesis has been investigated to date.

**Materials and Methods:**

An m6A mRNA epitranscriptomic microarray analysis was performed to analyze m6A regulator expression patterns and m6A methylation patterns in immune cells of T1DM patients (n=6) and healthy individuals (n=6). A bioinformatics analysis was subsequently performed to explore the potential biological functions and signaling pathways underlying T1DM pathogenesis. Furthermore, mRNA expression and m6A methylation levels were subsequently verified by qRT–PCR and methylated RNA immunoprecipitation–qPCR (MeRIP–qPCR), respectively, in the T1DM and healthy groups (n=6 per group).

**Results:**

Among the multiple m6A regulators, METTL3 and IGF2BP2 had significantly downregulated expression, and YTHDC1 and HNRNPA2B1 had significantly upregulated expression in the T1DM group relative to the healthy group. The microarray analysis revealed 4247 differentially methylated transcripts, including 932 hypermethylated and 3315 hypomethylated transcripts, and 4264 differentially expressed transcripts, including 1818 upregulated transcripts and 2446 downregulated transcripts in the T1DM group relative to the healthy group. An association analysis between methylation and gene expression demonstrated that the expression of 590 hypermethylated transcripts was upregulated, and that of 1890 hypomethylated transcripts was downregulated. Pearson correlation analysis showed significant correlations between the expression levels of differentially expressed m6A regulators and the methylation levels of differentially methylated transcripts and significant correlations between the expression levels of differentially expressed m6A regulators and that of differentially expressed transcripts. Moreover, Gene Ontology (GO) and Kyoto Encyclopedia of Genes and Genomes (KEGG) pathway analyses demonstrated that differentially methylated transcripts were involved in pathways related to immunity, including some closely associated with T1DM.

**Conclusions:**

Our study presents m6A regulator expression patterns and m6A methylation patterns of immune cells in T1DM, showing that the m6A mark and m6A regulators are promising targets for T1DM diagnosis and treatment.

## Introduction

Type 1 diabetes mellitus (T1DM), a chronic autoimmune disease, is characterized by absolute insulin deficiency. Complications of T1DM are classified into acute and chronic complications. The former mainly refers to ketoacidosis, while the latter includes cardiovascular diseases, kidney diseases, neuropathy and retinopathy, which may contribute to disability and death (1, 2). Insulin replacement therapy has been the only treatment for T1DM for a long time, and it can, indeed, save the lives of T1DM patients. However, insulin replacement therapy cannot prevent the development of long-term complications and can cause life-threatening hypoglycemia (2). Therefore, effective approaches for preventing T1DM progression or curing T1DM are urgently needed. However, the details of T1DM pathogenesis remain incompletely understood.

In T1DM, pancreatic β cells are destroyed by the innate and adaptive immune system, leading to decreased insulin secretion and hyperglycemia. A variety of immune cells participate in the destruction of β cells, including T cells, B cells, dendritic cells (DCs), and macrophages (3, 4). T cells play an extremely significant role in the formation of immune tolerance mechanisms. In the bone marrow, hematopoietic stem cells differentiate into T-cell progenitors, migrate to the thymus and undergo a negative selection process (3). In this process, T cells that bind too strongly to autoantigens are cleared. The surviving T cells then circulate in the peripheral blood and lymph nodes, where they can recognize and bind to the corresponding peptide complex. In T1DM, autoreactive T cells that specifically target β cells are preserved due to a breakdown in self-tolerance (3). β-cell peptides are thus presented to autoreactive CD4+ T cells by antigen-presenting cells (mainly DCs and B cells), leading to the activation of autoreactive CD8+ T cells in lymph nodes (5). Then, the activated CD8+ T cells migrate to islets and destroy β cells. B cells produce islet-specific autoantibodies, and innate immune cells release proinflammatory cytokines and reactive oxygen species, further activating the immune response and exacerbating β-cell destruction (5). Moreover, regulatory T cells (Tregs) maintain a noninflammatory environment by suppressing the proliferation and cytokine response of other immune cells (4). Therefore, Treg defects can aggravate the destruction of β cells.

N6-methyladenosine (m6A) acts as a new area of research in the epitranscriptome field that is contributing to medical advancements. m6A regulators consist of writers, erasers and readers that mediate the dynamic regulation of m6A formation and corresponding functions (6, 7). Writers are methyltransferase complexes composed of methyltransferase-like 3 (METTL3), the only one active catalytic subunit, and many auxiliary subunits: methyltransferase-like 14 (METTL14), Wilms tumor 1-associated protein (WTAP), Vir-like m6A methyltransferase associated (VIRMA)//KIAA1429, RNA binding motif protein 15 (RBM15), RNA binding motif protein 15B (RBM15B), methyltransferase-like 16 (METTL16), Casitas B-lineage lymphoma-transforming sequence-like protein 1 (CBLL1)/HAKAI, zinc finger CCCH domain-containing protein 13 (ZC3H13) and zinc finger CCHC-type containing 4 (ZCCHC4) (6, 7).Writers are responsible for installing m6A at specific sites within certain regions. Erasers, which mainly include fat mass and obesity-associated protein (FTO)/ALKB homolog 9 (ALKBH9) and ALKB homolog 5 (ALKBH5), are demethylases responsible for the removal of the m6A mark. Opposite processes are catalyzed by writers and erasers to maintain a balance between nonmethylated adenosine and m6A in RNA. Readers are RNA-binding proteins that recognize and combine with m6A sites to exert regulatory roles in the fate determination of target mRNAs and their biological functions. According to their interaction patterns, readers are classified into direct and indirect readers and consist of YTH domain-containing family protein 1-3 (YTHDF1-3), YTH domain-containing protein 1-2 (YTHDC1-2), insulin-like growth factor 2 binding protein 1-3 (IGF2BP1-3), heterogeneous nuclear ribonucleoprotein (HNRNP) A2B1, HNRNPC, HNRNPG and others (6, 7).

m6A is involved in all aspects of mRNA and noncoding RNA metabolism and activity, including alternative splicing, nuclear export, degradation, translation, microRNA biogenesis and circRNA expression (6, 8). By mediating gene expression, m6A participates in varieties of physiological and pathological processes: development of multiple systems, energy homeostasis, circadian rhythms, the occurrence and progression of cancer and cardiovascular diseases and other processes (6, 9–11). Recent evidence has suggested that m6A levels and m6A regulator expression levels are altered in autoimmune diseases (12). For example, global m6A and METTL3 expression levels have been shown to be significantly increased, while FTO, ALKBH5 and YTHDF2 mRNA levels have been shown to be significantly downregulated in rheumatoid arthritis (RA) (13, 14). The expression levels of METTL3, METTL14, WTAP, FTO, ALKBH5 and YTHDF2 have been found to be decreased in systemic lupus erythematosus (15, 16). Moreover, METTL14 deficiency in T cells induced spontaneous colitis by preventing the differentiation of T cells (17). ALKBH5 deficiency in T cells inhibited the development of colitis and experimental autoimmune encephalomyelitis by suppressing T-cell migration and T-cell-mediated immune responses (18). These findings indicate the important role played by the m6A modification in the development of autoimmune disorders. However, the roles played by m6A and m6A regulators in the pathogenesis of T1DM remains unclear, and research directly addressing these factors in T1DM is lacking.

Peripheral blood mononuclear cells (PBMCs) constitute a population of immune cells that consist of T cells, B cells, DCs, monocytes and natural killer cells. In this study, we extracted total RNA from isolated PBMCs and performed an m6A mRNA epitranscriptomic microarray analysis to explore the m6A regulator expression patterns and m6A methylation patterns in immune cells of T1DM patients. Then, we screened differentially methylated or expressed mRNAs based on their fold changes (FC) and *p* values. Gene Ontology (GO) and Kyoto Encyclopedia of Genes and Genomes (KEGG) pathway analyses were conducted to predict the potential functions and molecular pathways underlying T1DM pathogenesis. Finally, the expression levels and methylation levels in the microarray results were validated by quantitative real-time PCR (qRT–PCR) and methylated RNA immunoprecipitation coupled with qRT–PCR (MeRIP–qPCR). Our data show distinct patterns in m6A regulator expression and m6A modification in immune cells between T1DM and healthy controls, providing insights into the role played by m6A in the development of T1DM.

## Materials and methods

### Subjects

The sample collection and related protocols were approved by the Ethics Committee of the National Clinical Research Center of Second Xiangya Hospital, Central South University (Reference Number: 2021-S050). All participants were informed and provided signed written informed consent before peripheral blood collection. All blood samples in our study were drawn from T1DM patients (n=6) and healthy controls (n=6) matched by age and sex (**Table 1**). T1DM patients who fulfilled all of the following criteria were recruited into the case group: (1) diagnosed with diabetes based on World Health Organization (WHO) guidelines published in 1999; (2) no acute onset or diabetic ketoacidosis; (3) undergoing insulin-dependent therapy within six months of diagnosis; (4) positive for at least one classic islet autoantibody; and (5) not pregnant, carrying malignant tumors, or diagnosed with other autoimmune diseases or other types of diabetes. Healthy individuals fulfilling all of the following criteria were recruited into the control group: (1) a normal oral glucose tolerance test (OGTT), mean fasting blood glucose level < 5.6 mmol/l and a 2 h blood glucose level < 7.8 mmol/l after oral glucose challenge; (2) not pregnant, carrying malignant tumors or diagnosed with chronic diseases such as an autoimmune disease or a cardiovascular disease; and (3) no family history of diabetes.

**Table 1:** General characteristics of study subjects.

Characteristics	Type 1 diabetes	Healthy controls	*p* value
Sex (male/female)	4/2	3/3	1.000
Age (year)	15.50(14.00-20.75)	22.00(20.75-25.25)	0.058
BMI (kg/m2)	20.99±3.40	22.13±3.28	0.568
FBG (mmol/L)	9.66±3.65	4.52±0.40	0.018*
PBG (mmol/L)	17.26±5.63	4.88±0.87	0.003**
HbA1c (%)	6.70 (6.58-8.30)	5.35 (4.95-5.50)	0.002**
FCP (pmol/L)	35.05 (16.50-230.45)	387.70 (303.25-531.43)	0.004**
PCP (pmol/L)	57.90 (16.50-348.53)	1194.95 (930.83-1759.38)	0.002**
TG (mmol/L)	0.71±0.23	1.25±0.62	0.075
TC (mmol/L)	3.74±0.51	4.67±0.83	0.042*
HDL-C (mmol/L)	1.32±0.12	1.51±0.27	0.160
LDL-C (mmol/L)	1.97±0.44	2.73±0.79	0.072
GADA postive (%)	100.00 (6/6)	–	–
IA-2A postive (%)	50.00 (3/6)	–	–
ZnT8A postive (%)	33.33 (2/6)	–	–

BMI, body mass index; FBG, fasting blood glucose; PBG, 2-hour postprandial blood glucose; FCP, fasting C-peptide; PCP, 2-hour postprandial C-peptide; TG, triglyceride; TC, total cholesterol; HDL-C, high density lipoprotein-cholesterol; LDL-C, low density lipoprotein-cholesterol; GADA, glutamic acid decarboxylase antibody; IA-2A, protein tyrosine phosphatase antibody; ZnT8A, zinc transporter 8 antibody. *p < 0.05, **p < 0.01.

### Total RNA extraction and quality control

Peripheral blood (5 mL) diluted with an equal volume of PBS was slowly added to Ficoll-Paque^TM^ PLUS (GE Healthcare, Little Chalfont, Buckinghamshire, UK, Cat.No.17144003) in a centrifuge tube. After centrifuging the sample for 25 min at 800 g, four layers were obtained: an upper yellow plasma layer, a white PBMC layer in the interface, a layer of Ficoll medium and a red blood cell layer. The PBMCs were gently removed and transferred to a fresh centrifuge tube with a Pasteur pipette. Specifically, we started aspirating PBMCs at the periphery by using a Pasteur pipette and then slowly moved the pipette tip over the entire interface; we then transferred the PBMCs to another centrifuge tube. Alternatively, the upper plasma layer can be first removed before removing PBMCs. After being washed with PBS and centrifuged for 10 min at 300 g, the isolated PBMCs were frozen in freezing medium with 10% DMSO and 90% FBS at -80°C and then stored in liquid nitrogen until RNA extraction. The number of PBMCs obtained from 5 mL peripheral blood ranged from 5x10^6^ to 1x10^7^. Total RNA was extracted from these PBMCs with TRIzol reagent (Invitrogen, Carlsbad, CA, USA, Cat.No.15596026). The purity and amount of total RNA (varying in concentration between 400 ng/μl and 1000 ng/μl) were detected with a NanoDrop ND-1000 spectrophotometer (Thermo Fisher Scientific, Waltham, MA, USA), and RNA integrity was evaluated by denaturing agarose gel electrophoresis (**Figure S1**).

### m6A immunoprecipitation and microarray hybridization

Total RNA (3-5 μg) and an m6A spike-in control mixture were immunoprecipitated with 2 μg anti-m6A rabbit polyclonal antibody (Synaptic Systems, Gottingen, Germany, Cat.No.202003), which was incubated with head-over-tail rotation at 4°C for 2 h. A total of 20 μl of Dynabeads™ M-280 Sheep Anti-Rabbit IgG suspension (Invitrogen, Carlsbad, CA, USA, Cat.No.11203D) per sample was blocked with 0.5% bovine serum albumin at 4°C for 2 h. The immunoprecipitated (IP) fraction containing m6A-modified RNA was eluted from the immunoprecipitated magnetic beads, while the supernatant (Sup) fraction containing the m6A-unmodified RNA was recovered from the centrifuged supernatant. Then, the IP and Sup RNAs were labeled with Cy5 and Cy3, respectively, referred to as cRNAs by using Arraystar Super RNA Labeling Kit (Arraystar, Rockville, MD, USA, Cat.No.AL-SE-005). These cRNAs labeled with fluorescent dye were merged and hybridized in Human Arraystar mRNA&lncRNA Epitranscriptomic Arrays (8x60K, Arraystar) that contained 44,122 mRNAs and 12,496 lncRNAs. The arrays were washed and then detected with an Agilent scanner G2505C (Agilent Technologies, Santa Clara, CA, USA).

### m6A mRNA epitranscriptomic microarray analysis

The acquired array images were imported into Agilent Feature Extraction software (version 11.0.1.1), and the raw data were extracted. The raw intensities of the Cy5-labeled IP and Cy3-labeled Sup samples were normalized with the average of the log2-scaled spike-in control RNA intensity. “m6A quantity” was calculated to represent the m6A methylation level of each transcript based on the normalized intensity of the Cy5-labeled IP sample. The percentage of transcripts with m6A modification was calculated based on the normalized intensities of both the Cy5-labeled IP and Cy3-labeled Sup samples. The expression levels of the mRNAs were calculated based on the total normalized intensities of Cy5-labeled and Cy3-labeled RNAs.

The FC value and statistical significance of the difference (*p* value) for each transcript were calculated based on the m6A quantity and mRNA expression levels, and these data were used to compare the case and control groups. mRNAs with different m6A methylation levels between the two groups were determined according to the FC and *p* thresholds of m6A quantity (FC ≥ 2 or ≤ 0.5 and *p* < 0.05). mRNAs with differential expression between the two groups were determined according to the FC and *p* thresholds of expression level (FC ≥ 2 or ≤ 0.5 and *p* < 0.05). Hierarchical clustering analyses of differentially m6A-methylated RNAs and differentially expressed RNAs were performed with R software, including the gplots (version 2.16.0) and RColorBrewer (version 1.1.2) packages, and the results showed the distinct methylation and expression patterns among the samples.

### Correlation analysis of m6A regulators and differentially expressed or methylated transcripts

Pearson correlation analysis was performed to analyze the correlations between differentially expressed m6A regulators and both differentially expressed transcripts and differentially methylated transcripts. The Pearson correlation coefficient (PCC), *p* value and false discovery rate (FDR) were calculated based on the expression levels of m6A regulators and the mRNA expression or m6A methylation levels of differential transcripts. The records fulfilling |PCC| > 0.9, *p* value < 0.05, and FDR <1 were selected to be visualized in Cytoscape (version 3.8.1). A positive PCC value indicates a positive correlation, while a negative PCC value indicates a negative correlation.

### qRT–PCR

Based on the microarray results, published literature and m6A2Target database (http://m6a2target.canceromics.org/), we selected 5 m6A regulators and 5 immunity-related genes and confirmed their expression levels. Reverse transcription of the total RNA extracted from the PBMCs to generate cDNA was performed using a PrimeScript RT reagent kit (TaKaRa, Tokyo, Japan, Cat.No.RR037A). We prepared the Arraystar SYBR® Green qPCR Master Mix (Arraystar, Rockville, MD, USA, Cat.No.AS-MR-006-5) in each tube and added the RNA samples to the tubes containing Master Mix on ice. The tubes were incubated at 37°C for 15 min for reverse transcription and at 85°C for 5 s for heat inactivation; the tubes were then chilled at 4°C. Then, qRT–PCR was performed on a ViiA 7 Real-time PCR System (Applied Biosystems, Foster City, CA, USA) according the following PCR program: an initial 50°C incubation step for 2 min, a 95°C incubation step for 10 min and then 40 cycles of 95°C for 15 s and 60°C for 60 s. β-Actin was used as an internal standard. Three replicates of each sample were tested. Relative mRNA expression levels were normalized to β-actin expression in each reaction. The expression levels were calculated by the 2^−ΔΔCT^ method. The sequences of all the primers used in this study are listed in **Table S1**.

### MeRIP–qPCR

MeRIP–qPCR was performed to further validate the m6A methylation levels of the immunity-related genes identified as differentially expressed by qRT–PCR. Briefly, total RNA was fragmented and then divided into two parts: a large part and a small part. The large amount of fragmented RNA was immunoprecipitated with anti-m6A antibody, while the small amount was saved as input RNA without immunoprecipitation. The IP sample RNAs and input RNA were then subjected to RT–qPCR analysis according the experimental procedure described above. Three replicates of each sample were tested. The data were normalized on the basis of the input RNA. The sequences of the primers used in the experiment are listed in **Table S1**.

### GO and KEGG pathway analyses

The GO database (http://www.geneontology.org) describes the function of genes in three categories, and the KEGG database (http://www.genome.jp/kegg/) is used to annotate gene functions and identify complicated signaling pathways in which the related genes are expressed. The TopGO package (version 2.42.0) in the R environment was used to compute and graph the GO analysis data, while ingenuity pathway analysis was performed with the KEGG pathway results. GO and KEGG pathway analyses were carried out to fully elucidate the functions of differentially m6A-methylated mRNAs and identify the possible molecular pathways involved in T1DM. The -log10(*p* value) was transformed as the enrichment score value for a function or pathway. A *p* value <0.05 or an enrichment score >1.301 indicates that the function or pathway is of great significance to the physiological or pathological process of interest. A lower *p* value or higher enrichment score indicates a greater significance of differentially m6A-methylated mRNAs in relation to a function or pathway.

### Statistical analysis

IBM SPSS Statistics version 26.0, GraphPad Prism 7 and Cytoscape (version 3.8.1) were used to perform the statistical analyses and generate figures. An unpaired two-sided t test was applied to calculate the significance of differences between two groups in the microarray, RT–qPCR and MeRIP–qPCR analyses. Correlations were tested by Pearson correlation analysis. For the GO and KEGG pathway analyses, the statistical significance of the enrichment data was calculated by two-sided Fisher’s exact test and transformed into a -log10(*p* value) score that represents the enrichment. *p* < 0.05 was considered to be significant.

## Results

### Expression characteristics of m6A regulators

To explore the expression patterns of m6A regulators between T1DM and healthy individuals, we detected the expression levels of 23 major regulators, including 10 writers (METTL3, METTL14, WTAP, RBM15, RBM15B, VIRMA/KIAA1429, METTL16, CBLL1/HAKAI, ZC3H13, and ZCCHC4), 2 erasers (FTO/ALKBH9 and ALKBH5) and 11 readers (YTHDF1, YTHDF2, YTHDF3, YTHDC1, YTHDC2, IGF2BP1, IGF2BP2, IGF2BP3, HNRNPA2B1, HNRNPC, and HNRNPG). The microarray results showed that the expression levels of METTL3 and IGF2BP2 were significantly downregulated, while the expression levels of YTHDC1, HNRNPA2B1 and HNRNPC were significantly upregulated in T1DM patients. The expression levels of other regulators were not significantly different between the two groups (**Table 2**).

**Table 2:** The mRNA levels of m6A regulators detected by microarray analysis.

Gene symbol	Transcript ID	Fold change	Regulation	*p* value
METTL3	ENST00000298717	0.43	Down	0.0000
METTL14	ENST00000388822	1.87	Up	0.0193
WTAP	ENST00000631126	1.46	Up	0.2122
	ENST00000337387	1.00	Up	0.9982
RBM15	ENST00000618772	1.66	Up	0.0507
	ENST00000487146	1.26	Up	0.2850
RBM15B	ENST00000563281	1.55	Up	0.1211
VIRMA/KIAA1429	ENST00000421249	0.78	Down	0.0067
	ENST00000297591	1.35	Up	0.2041
METTL16	ENST00000263092	0.94	Down	0.6107
CBLL1/ HAKAI	ENST00000440859	0.68	Down	0.0317
	ENST00000222597	1.23	Up	0.5672
ZC3H13	NM_001330564	1.36	Up	0.0115
	NM_001330566	1.15	Up	0.4082
	ENST00000242848	1.42	Up	0.0168
ZCCHC4	–	–	–	–
ALKBH5	ENST00000399138	0.89	Down	0.4627
FTO/ALKBH9	ENST00000471389	0.97	Down	0.7877
YTHDF1	ENST00000370339	1.68	Up	0.0005
YTHDF2	ENST00000541996	1.08	Up	0.5172
YTHDF3	ENST00000517371	1.33	Up	0.1926
	ENST00000621413	1.61	Up	0.0834
	ENST00000623280	1.74	Up	0.0566
YTHDC1	ENST00000579690	1.56	Up	0.1666
	ENST00000344157	1.78	Up	0.2168
	ENST00000355665	2.06	Up	0.0307
YTHDC2	ENST00000161863	1.15	Up	0.6308
IGF2BP1	–	–	–	–
IGF2BP2	ENST00000421047	0.22	Down	0.0000
	ENST00000346192	1.05	Up	0.8094
	NM_001291872	1.38	Up	0.0758
	ENST00000457616	1.30	Up	0.2540
IGF2BP3	–	–	–	–
HNRNPA2B1	ENST00000354667	2.45	Up	0.0010
	ENST00000356674	2.48	Up	0.0007
HNRNPC	ENST00000553300	2.30	Up	0.0001
	ENST00000554969	2.09	Up	0.0048
	ENST00000554455	1.53	Up	0.1876
	ENST00000430246	0.98	Down	0.7724
	ENST00000557201	1.55	Up	0.0002
HNRNPG	–	–	–	–

METTL3/14/16, methyltransferase-like 3/14/16; WTAP, Wilms tumor 1-associated protein; RBM15/15B, RNA binding motif protein 15/15B; VIRMA, Vir-like m6A methyltransferase associated; CBLL1, Casitas B-lineage lymphoma-transforming sequence-like protein 1; ZC3H13, zinc finger CCCH domain-containing protein 13; ZCCHC4, zinc finger CCHC-type containing 4; ALKBH5, ALKB homolog 5; FTO, fat mass and obesity-associated protein; ALKBH9, ALKB homolog 9; YTHDF1/2/3, YTH domain-containing family protein 1/2/3; YTHDC1/2, YTH domain-containing protein 1/2; IGF2BPs, insulin-like growth factor 2 binding proteins; HNRNPA2B1/C/G, heterogeneous nuclear ribonucleoprotein A2B1/C/G.

### Identification of differentially methylated or expressed transcripts

**Figure 1:**
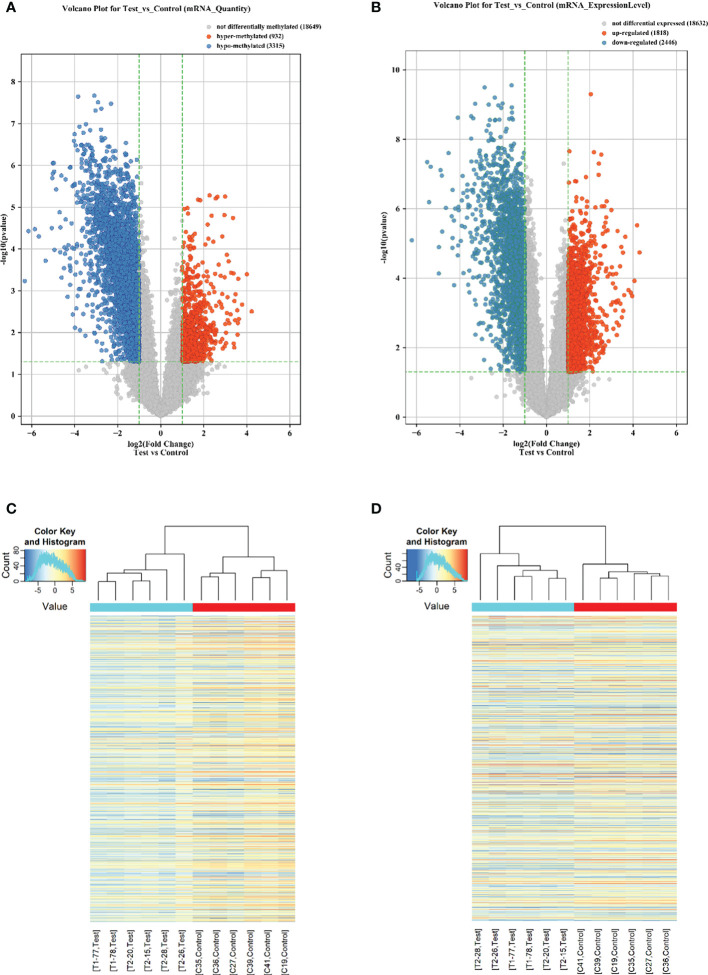
Overview of the m6A methylation landscape and mRNA expression profile. **(A)** Volcano plot showing differentially m6A-methylated transcripts. The blue boxes represent hypomethylated transcripts (FC ≤ 0.5, *p* < 0.05). The red boxes represent hypermethylated transcripts (FC ≥ 2, *p* < 0.05). **(B)** Volcano plot showing differentially expressed transcripts. The blue boxes represent downregulated transcripts (FC ≤ 0.5, *p* < 0.05). The red boxes represent upregulated transcripts (FC ≥ 2, *p* < 0.05). **(C)** Hierarchical clustering revealing a distinct mRNA methylation pattern between the T1DM group and the control group. **(D)** Hierarchical clustering revealing a distinct mRNA expression pattern between the T1DM group and the control group.

To investigate the differences in the m6A levels of the transcripts between the T1DM and control samples, we profiled the immunoprecipitated m6A-methylated RNAs. The microarray results demonstrated that 4247 transcripts were differentially methylated in T1DM samples relative to control samples, including 932 hypermethylated mRNAs and 3315 hypomethylated mRNAs (FC ≥ 2 or ≤ 0.5, *p* < 0.05) (**Figure 1A**; **Table S2**). In addition, we profiled total mRNAs by analyzing a microarray to investigate the mRNA expression patterns. The microarray profiling analysis showed that a total of 4264 mRNAs were significantly differentially expressed in T1DM compared with controls (FC ≥ 2 or ≤ 0.5, *p* < 0.05) (**Figure 1B**). The expression of 1818 mRNAs was significantly upregulated, while that of 2446 mRNAs was significantly downregulated (**Figure 1B**; **Table S2**). Hierarchical clustering identified the interrelationships between the samples and showed distinct m6A methylation patterns and expression patterns of the mRNAs between the T1DM group and the healthy group (**Figures 1C, D**).

### Interaction between m6A methylation and mRNA expression

**Figure 2:**
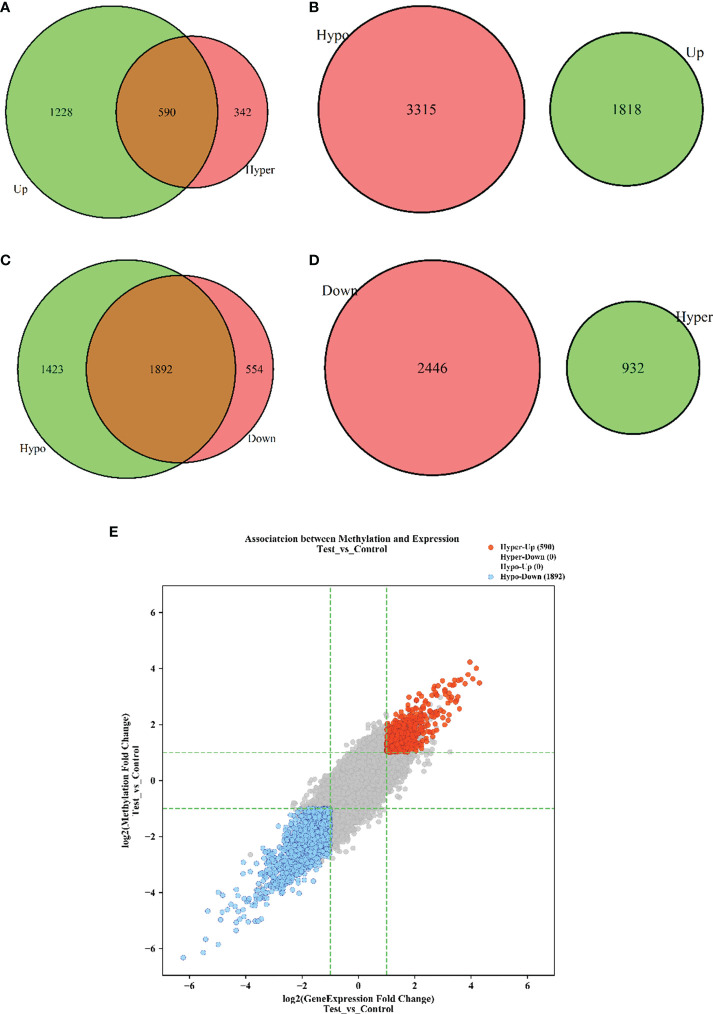
The interaction between m6A methylation and mRNA expression. **(A)** Hypermethylation-Upregulation. **(B)** Hypomethylation-Upregulation. **(C)** Hypomethylation-Downregulation. **(D)** Hypermethylation-Downregulation. The red circles and green circles represent differentially expressed mRNAs or differentially methylated mRNAs, and the brown regions represent the mRNAs with significant differences in both methylation level and expression level. **(E)** Four-quadrant diagram of transcripts with significant differences in both m6A methylation and mRNA expression. The red dots represent hypermethylated-upregulated mRNAs, while the blue dots represent hypomethylated-downregulated mRNAs. *p* < 0.05.

**Figure 3:**
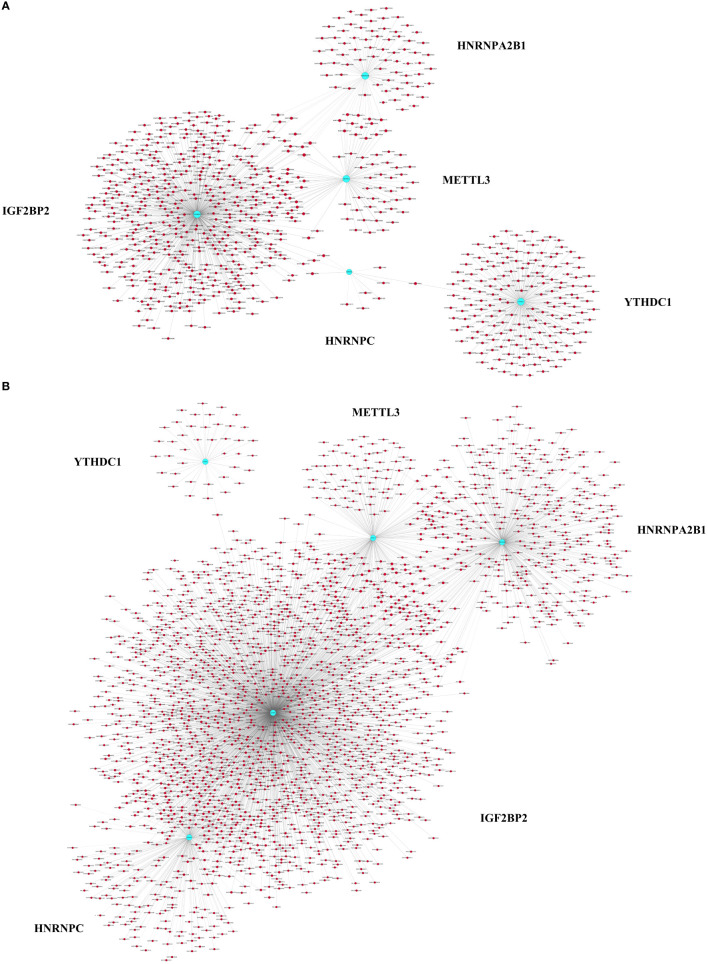
Pearson correlation analysis of m6A regulators and differentially expressed or methylated transcripts. **(A)** Correlations between the expression levels of m6A regulators and the methylation levels of differentially methylated transcripts. **(B)** Correlations between the expression levels of m6A regulators and the expression levels of differentially expressed transcripts. The blue nodes represent m6A regulators, and the red nodes represent differentially methylated or expressed transcripts. |PCC| > 0.9, FDR <1, *p* < 0.05.

To explore whether m6A methylation influences mRNA translation, we identified the intersection of differentially expressed mRNAs and differentially methylated m6A mRNAs and performed an association analysis between the methylation and expression results. Surprisingly, only two modes of interaction were identified. The expression of 590 hypermethylated mRNAs was upregulated, and that of 1890 hypomethylated mRNAs was downregulated (**Figures 2A, C, E**; **Table S3**). However, no intersection between hypermethylation and downregulation or hypomethylation and upregulation was observed (**Figures 2B, D, E**). Hypomethylated mRNAs with downregulated expression accounted for the largest proportion of methylated RNAs in T1DM. These results showed that hypermethylation is accompanied by upregulated mRNA expression, while hypomethylation is accompanied by downregulated mRNA expression. Furthermore, we analyzed the correlations between differentially expressed m6A regulators and differentially methylated or expressed transcripts. There were strong correlations between the expression levels of m6A regulators and the methylation levels of differentially methylated transcripts and significant correlations between the expression levels of m6A regulators and the expression levels of differentially expressed transcripts (**Table S4**; **Figure 3A, B**). These results indicated that m6A regulators, especially IGF2BP2, may impact mRNA expression in an m6A-dependent manner.

### Potential functions and pathways of differentially methylated transcripts

**Figure 4:**
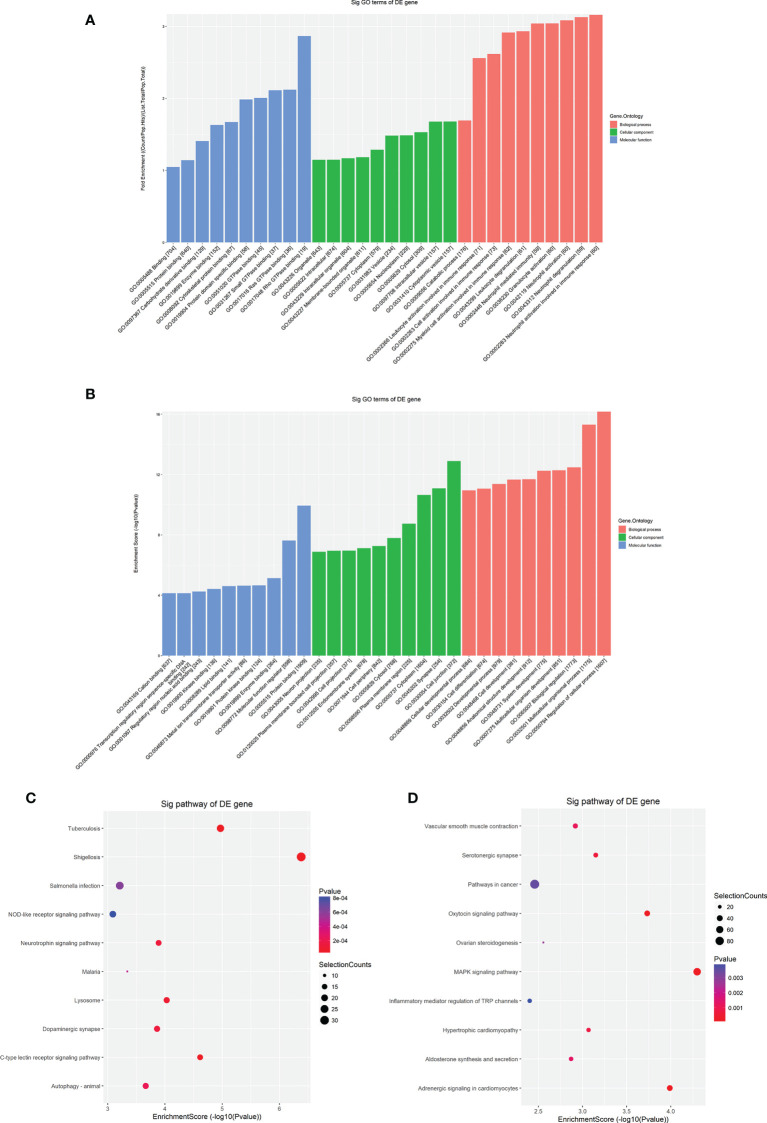
GO and KEGG pathway analyses of differentially methylated mRNAs. **(A)** The top ten enriched GO items of differentially hypermethylated mRNAs. **(B)** The top ten enriched GO items of differentially hypomethylated mRNAs. **(C)** The top ten enriched pathways of differentially hypermethylated mRNAs. **(D)** The top ten enriched pathways of differentially hypomethylated mRNAs.

To explore the potential biological functions and molecular pathways underlying the m6A modification, we carried out GO and KEGG pathway analyses of differentially m6A-methylated transcripts. Hypermethylated mRNAs were enriched in 1418 GO terms and 62 KEGG pathways (**Table S5**). The GO analysis showed that hypermethylated mRNAs were widely distributed in the cytoplasm, organelles and vesicles and were involved in immunity activation and metabolic processes by binding proteins and energy-related components (**Figure 4A**). The KEGG analysis showed that hypermethylated mRNAs were primarily associated with pathogen infection, immune cell differentiation, lysosomes, autophagy and neural signal transmission (**Figure 4C**). Moreover, hypermethylated mRNAs were involved in cancer pathways, the B-cell receptor signaling pathway and energy metabolism (**Table S5**). These results indicated that high m6A methylation may contribute to the pathogenesis of immunity-related diseases, nervous diseases and metabolic diseases.

Hypomethylated mRNAs were found to be enriched in 1632 GO terms and 47 KEGG pathways (**Table S6**). The GO analysis showed that hypomethylated mRNAs were mainly distributed in the cytoplasm, endomembrane system and components critical for conveying information and communicating (**Figure 4B**). These mRNAs participated in the regulation of biological processes and developmental processes by binding multiple signaling molecules and regulators (**Figure 4B**). The KEGG analysis showed that hypomethylated mRNAs were primarily associated with cellular regulation, cancers, inflammation, cardiovascular function, and multiple processes involving hormones, including synthesis, secretion and signaling (**Figure 4D**). In addition, hypomethylated mRNAs were involved in material metabolism, development, pathogen infection and signal transmission in the nervous system (**Table S6**). These results indicated that low m6A methylation may contribute to the pathogenesis of disease in multiple systems including the immune system, nervous and mental system, cardiovascular system and endocrine system.

**Figure 5:**
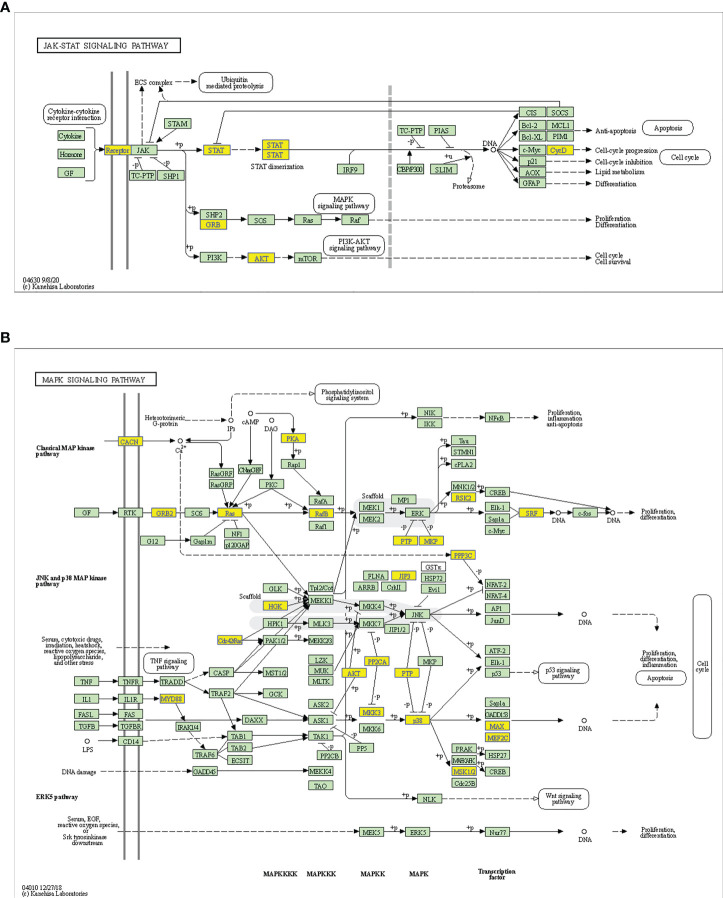
Schematic overviews of T1DM-related signaling pathways enriched with hypermethylated mRNAs. **(A)** JAK-STAT signaling pathway. **(B)** MAPK signaling pathway. The yellow nodes represent hypermethylated mRNAs. The green nodes represent other mRNAs in the pathways that are not regulated by m6A.

**Figure 6:**
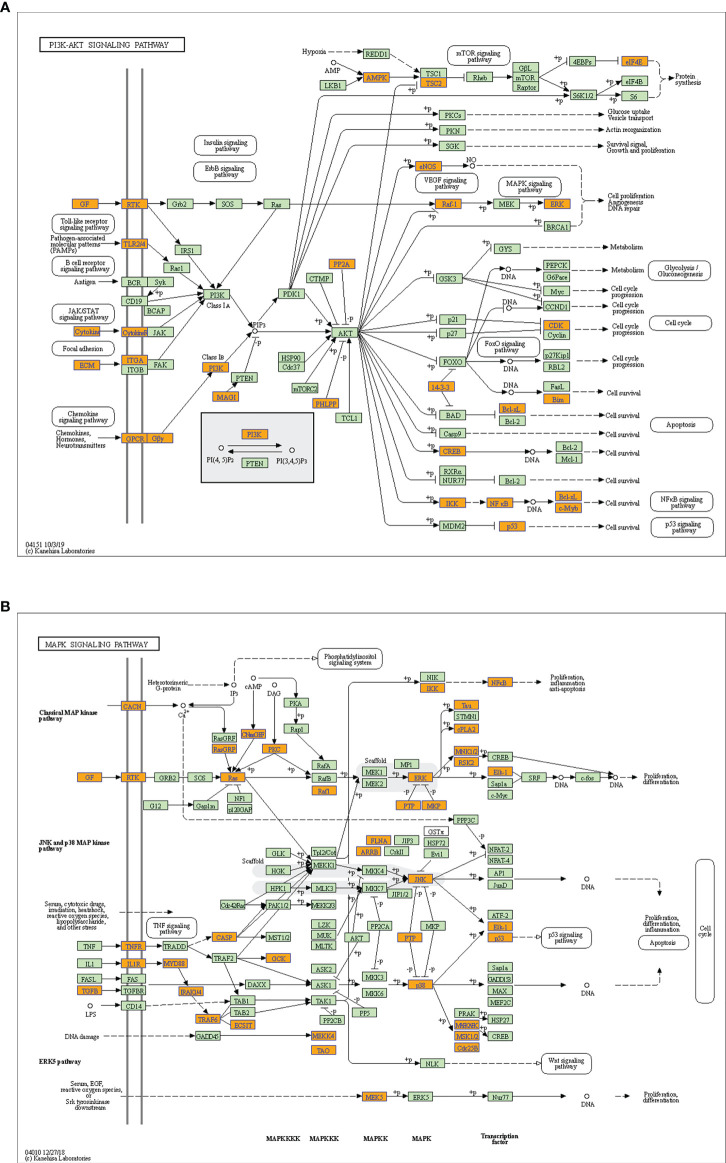
Schematic overviews of T1DM-related signaling pathways enriched with hypomethylated mRNAs. **(A)** PI3K-Akt signaling pathway. **(B)** MAPK signaling pathway. The orange nodes represent hypomethylated mRNAs. The green nodes represent other mRNAs in the pathways that are not regulated by m6A.

Considering these results, m6A balance is essential for normal physiological processes. Aberrant m6A levels, including either higher or lower levels, cause the development of diverse diseases. In addition, T1DM-related pathways mainly consist of cytokine–cytokine receptor interactions, the JAK-STAT signaling pathway, the PI3K-Akt signaling pathway, the MAPK signaling pathway, and antigen processing and presentation. Our results showed that hypermethylated transcripts were enriched in the JAK-STAT signaling pathway and MAPK signaling pathway, while hypomethylated transcripts were enriched in the PI3K-Akt signaling pathway and MAPK signaling pathway, indicating the potential roles of m6A in the pathogenesis of T1DM (**Figures 5A, B**, **6A, B**).

### Validation of key regulators and immunity-related genes by qRT–PCR and MeRIP–qPCR

**Figure 7:**
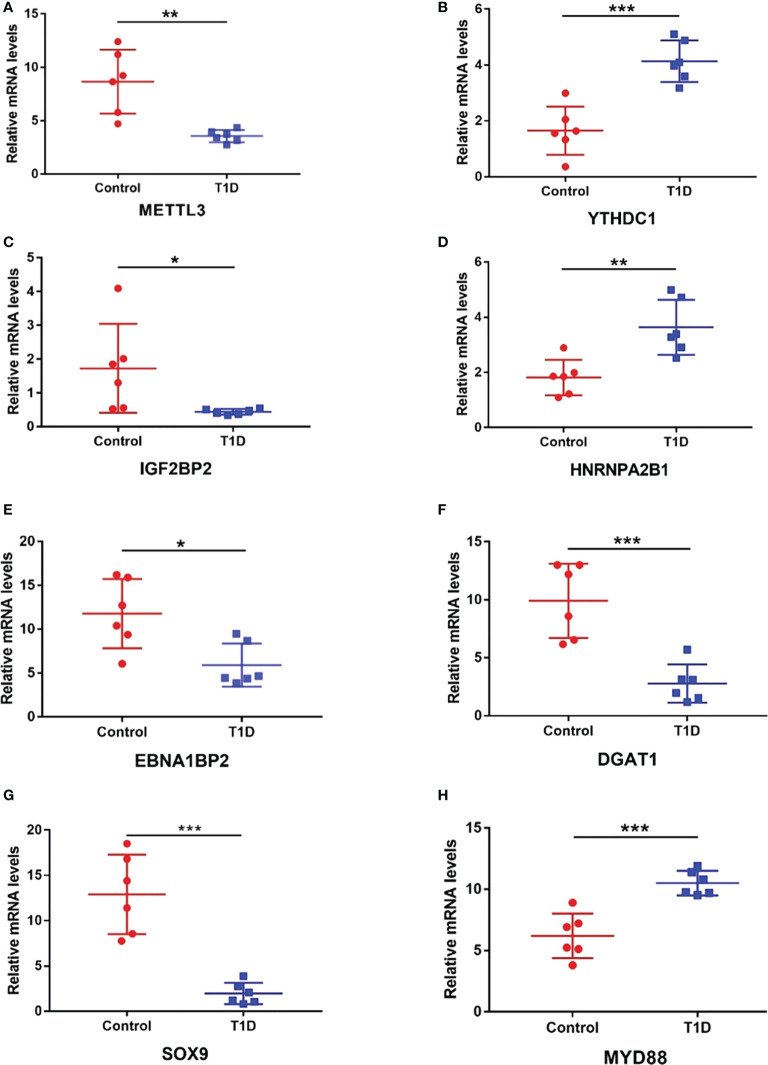
The expression levels of m6A regulators and key immunity-related genes verified by RT–qPCR. **(A)** METTL3. **(B)** YTHDC1. **(C)** IGF2BP2. **(D)** HNRNPA2B1. **(E)** EBNA1BP2. **(F)** DGAT1. **(G)** SOX9. **(H)** MYD88. n=6 each group, *p < 0.05, **p < 0.01, and ***p < 0.001.

**Figure 8:**
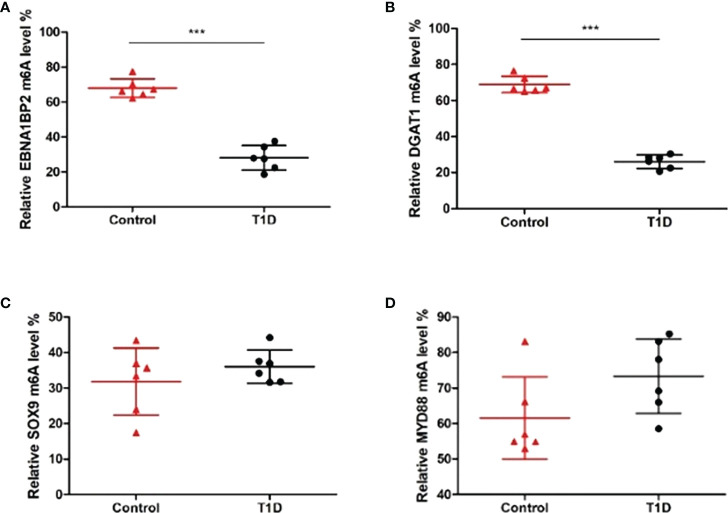
The m6A methylation levels of key immunity-related genes verified by MeRIP–qPCR. **(A)** EBNA1BP2. **(B)** DGAT1. **(C)** SOX9. **(D)** MYD88. n=6 per group, ***p < 0.001.

Considering the microarray results, we chose 5 m6A regulators (METTL3, YTHDC1, IGF2BP2, HNRNPA2B1 and HNRNPC) (FC ≥ 2 or ≤ 0.5 and *p* < 0.05) and verified their expression levels by qRT–PCR (**Table 2**). qRT–PCR was also performed to verify the expression levels of 5 key immunity-related genes selected based on the microarray results, published literature and m6A2Target database (**Table 3**). We particularly focused on the role of METTL3 and extracted all validated targets of METTL3 based on the m6A2Target database. Then, we selected the overlapping genes of validated targets and differentially expressed genes (FC ≥ 2 or ≤ 0.5 and *p* < 0.05) with downregulated m6A levels (FC ≤ 0.5 and *p* < 0.05) in the microarray results, and we selected the top 20 genes with decreased m6A levels and differential expression. We further screened 5 immunity-related genes according to the literature: downregulated EBNA1-binding protein 2 (EBNA1BP2) (19), diacylglycerol O-acyltransferase-1 (DGAT1) (20), sex determining region Y-box 9 (SOX9) (21), SP4 (22) and upregulated myeloid differentiation factor 88 (MYD88) (23). The qRT–PCR results showed that the differences in METTL3, YTHDC1, IGF2BP2, HNRNPA2B1, EBNA1BP2, DGAT1, SOX9 and MYD88 expression were statistically significant, in accordance with the microarray analysis, while differences in HNRNPC and SP4 expression were not significant (**Figures 7A-H**). Furthermore, the m6A modification levels of EBNA1BP2, DGAT1, SOX9 and MYD88 were verified by MeRIP–qPCR (**Table 4**). MeRIP–qPCR analysis showed that only EBNA1BP2 and DGAT1 were significantly hypomethylated and that SOX9 and MYD88 were not significantly hypermethylated (**Figures 8A-D**).

**Table 3:** The expression levels of key immunity-related genes in the microarray analysis.

Gene symbol	Transcript ID	Fold change	Regulation	*p* value
EBNA1BP2	uc001cio.3	0.01	Down	0.0000
DGAT1	ENST00000528718	0.08	Down	0.0001
SOX9	ENST00000245479	0.06	Down	0.0000
SP4	ENST00000222584	0.40	Down	0.0002
MYD88	ENST00000417037	2.73	Up	0.0119

EBNA1BP2, EBNA1-binding protein 2; DGAT1, diacylglycerol O-acyltransferase-1; SOX9, sex determining region Y-box 9; MYD88, upregulated myeloid differentiation factor 88.

**Table 4:** The m6A methylation levels of key immunity-related genes in the microarray analysis.

Gene symbol	Transcript ID	Fold change	Regulation	*p* value
EBNA1BP2	uc001cio.3	0.01	Hypo	0.0006
DGAT1	ENST00000528718	0.03	Hypo	0.0000
SOX9	ENST00000245479	0.04	Hypo	0.0000
SP4	ENST00000222584	0.18	Hypo	0.0000
MYD88	ENST00000417037	2.27	Hyper	0.0223
	ENST00000443433	2.62	Hyper	0.0241
	ENST00000396334	0.46	Hypo	0.0147

## Discussion

In recent decades, m6A has become a hot topic in the scientific research field and provides novel approaches for the diagnosis and therapy of various diseases. Mounting evidence suggests that m6A methylation levels and the expression levels of m6A regulators are altered in autoimmune diseases and that these changes may contribute to the initiation and progression of autoimmune diseases. However, the potential role played by m6A in the development of T1DM had not been extensively studied. Therefore, in this study, we analyzed the expression patterns of m6A regulators and m6A methylation patterns in immune cells obtained from T1DM patients.

Accumulating evidence has demonstrated that m6A and m6A regulators are crucial for the development, differentiation, activation and homeostasis of immune cells, indicating their potential roles in the development of autoimmune diseases (12). Our study demonstrated that a writer (METTL3) and 3 readers (HNRNPA2B1, IGF2BP2 and YTHDC1) were expressed at significantly different levels between T1DM and healthy samples. We paid considerable attention to the potential role of METTL3 in the development of T1DM. The METTL3 expression level was notably upregulated in the podocytes of patients with diabetic nephropathy and the kidneys of type 1 and type 2 diabetic mice. METTL3 modulates Notch signaling by catalyzing m6A modification of TIMP2 in an IGF2BP2-dependent manner, leading to proinflammatory and proapoptotic effects in podocytes (24). METTL3 expression was found to be substantially increased in the synovial tissues of RA patients and an RA rat model. Fibroblast-like synoviocytes (FLSs) play an essential role in RA pathogenesis by releasing proinflammatory indicators and penetrating the synovial membrane. METTL3 may not only mediate the expression of proinflammatory cytokines in FLSs but may also promote the activation, proliferation, invasion and migration of FLSs as mediated by the NF-κB signaling pathway, ultimately accelerating RA development (14). Another study also showed that METTL3 expression was significantly increased in the PBMCs of RA patients. However, the authors came to an opposite conclusion that METTL3 inhibited the macrophage-mediated inflammatory response through the NF-κB signaling pathway under lipopolysaccharide stimulation (25). These results indicate that METTL3 may be a promising candidate target for T1DM therapy.

To further explore the role played by m6A in gene expression, we performed an association analysis between m6A quantity and mRNA expression level. Surprisingly, the association analysis showed only two modes of interaction between differentially methylated mRNAs and differentially expressed mRNAs: hypermethylation-upregulation and hypomethylation-downregulation. The reason why no hyper-down or hypo-up transcripts were identified in our study may be due to that higher FC threshold was chosen for finding more significantly differentially methylated or expressed transcripts. The FC value of all hypermethylated or upregulated transcripts were ≥2, while the FC value of all hypomethylated or downregulated transcripts were ≤0.5 in the study.

In our study, the changes in mRNA expression level were consistent with those in m6A level, demonstrating that increases in m6A levels are accompanied by upregulated gene expression, while decreases in m6A levels are accompanied by downregulated gene expression. This outcome may be attributable to reader involvement. IGF2BP2 can stabilize m6A-modified transcripts in an m6A-dependent manner (26). YTHDC1 has been reported to modulate alternative splicing, regulate nuclear export, accelerate the decay of m6A-modified transcripts and suppress gene expression (27–29). HNRNPA2B1 can also modulate alternative splicing of target transcripts by recognizing and binding to m6A on the basis of a switch mechanism (30). Therefore, IGF2BP2, YTHDC1 and HNRNPA2B1 may be involved in regulating mRNA expression levels in an m6A-dependent manner in T1DM; this possibility is consistent with the correlation analysis results. However, the detailed interaction and precise molecular mechanisms need to be further investigated.

We then performed GO and KEGG pathway analyses to predict the potential biological functions and identify the signaling pathways enriched with differentially methylated mRNAs. The GO and KEGG analysis showed that differentially methylated mRNAs were involved in immunity-related pathways. Most importantly, we found that both hypermethylated transcripts and hypomethylated transcripts were enriched in T1DM-related pathways, including the JAK-STAT, PI3K-Akt and MAPK signaling pathways. These signaling pathways are involved in the proliferation, development, differentiation and cytokine production of immune systems, mediating autoimmune responses in T1DM (31, 32). Based on these findings, m6A may participate in T1DM pathogenesis through immunity-related pathways.

Notably, we found that both hypermethylated mRNAs and hypomethylated mRNAs were enriched in some biological functions and KEGG terms, indicating that aberrant m6A levels, i.e., hypermethylation or hypomethylation, can result in the development of some diseases. This phenomenon can be attributed to the following reasons. Hyper- and hypomethylated mRNAs can participate in the same biological function by activating different signaling molecules in the same or different pathways. In addition, some biological functions are regulated by opposing pathways. Therefore, hyper- and hypomethylated mRNAs can participate in the pathogenesis of the same disease by promoting pathogenic pathways and inhibiting protective pathways, respectively. Moreover, we found that the MAPK signaling pathway, the T1DM-related pathway, was represented in the enrichment results of both hyper- and hypomethylated transcripts. This finding indicates that hyper- and hypomethylated transcripts may activate this pathway by recruiting different reader proteins and subsequently activating different signaling molecules.

Specifically, we chose certain immunity-related genes to perform further analysis. Numerous studies have indicated that MYD88 activation plays a vital role in driving autoimmunity and promoting autoimmune disease progression (23, 33). Notably, MYD88 deficiency completely prevented NOD mice from developing T1DM in a microbiota-dependent manner (34). Our study showed that MYD88 expression was upregulated in T1DM patient samples compared to that in the control samples, which was consistent with previous studies and in accordance with the role it plays in the development of T1DM (35, 36). However, differences in the m6A level on MYD88 were not significantly different, indicating that MYD88 expression was mediated in an m6A-independent manner. The transcription factor SOX9 may be involved in the mechanism leading to the loss of β cells (not β-cell apoptosis) in T1DM. The changes to the identity of β cells due to dedifferentiation and/or transdifferentiation were accompanied by increased SOX9 expression in the β cells (37, 38). Moreover, SOX9+ ductal cells may differentiate into β cells, while SOX9+ progenitors may be dispensable for regenerating β cells in T1DM (39, 40). Therefore, SOX9 may represent a previously unidentified marker of human β-cell dedifferentiation and redifferentiation and is likely to become a new target for the therapy and reversal of T1DM. However, SOX9 expression was decreased in the T1DM samples in our study, possibly because the samples consisted of immune cells, not β cells.

In addition, DGAT1 inhibited regulatory T-cell formation by forming retinyl ester in a retinol-dependent manner, thus leading to an imbalance between effector T cells and Tregs. DGAT1 induced Treg formation to attenuate experimental autoimmune encephalomyelitis (20). However, our results showed that DGAT1 was hypermethylated and downregulated in T1DM, indicating that DGAT1 may not be a key player in T1DM development. EBNA1BP2 has been confirmed to be one of the hub genes in COVID-19 susceptibility and was related to the levels of infiltrating immune cells, including CD4+ activated memory T cells, follicular helper T cells and plasma cells (19). Moreover, EBNA1BP2 was identified as the most significantly downregulated transcript accompanied by hypomethylation in our microarray results. Whether EBNA1BP2 is involved in T1DM pathogenesis remains to be further investigated.

Although T1DM is still incurable, immune-based therapy may be an innovative, effective and acceptable method to treat and potentially reverse T1DM (3). However, a lack of effective and safe immune intervention targets has hindered therapy development. Over several decades, m6A has been a promising target for the diagnosis and treatment of all kinds of diseases. STM2457, the selective catalytic inhibitor of METTL3, has been found to reduce the growth of acute myeloid leukemia (AML) and promote differentiation and apoptosis, thus preventing and slowing AML progression in mouse models (41). These findings indicate that targeting RNA-modifying enzymes may be a promising strategy in disease therapy.

Studies on the role of m6A in the pathological process of T1DM are limited, and all to date have been focused on the role of m6A in the progression of T1DM complications. The present study is the first to provide a comprehensive overview of the m6A regulator expression and m6A methylation patterns of PBMCs in T1DM. However, there are limitations to the study. First, the sample size was relatively small. These findings need to be validated in analysis with more samples in future studies. Second, the specific functions and downstream targets of the m6A regulators in this study are unclear, although our correlation analysis revealed many potential targets of these m6A regulators. Further studies should concentrate on the specific targets of m6A regulators and the exact m6A mechanisms in the pathogenesis of T1DM by knocking out or overexpressing specific genes in cell and animal models.

In conclusion, our study identified the expression patterns of m6A regulators and m6A methylation patterns of immune cells in individuals with T1DM and healthy individuals and explored the potential biological functions and pathways associated with T1DM pathogenesis. Our study suggests that the m6A mark and related regulators may serve as biological markers and putative immune targets for the diagnosis, treatment and eventual reversal of T1DM.

## Data availability statement

The original contributions presented in the study are publicly available. This data can be found here: the Open Archive for Miscellaneous Data (OMIX, https://ngdc.cncb.ac.cn/omix) (Accession number: OMIX001855).

## Ethics statement

The studies involving human participants were reviewed and approved by the ethics committee of the National Clinical Research Center of Second Xiangya Hospital, Central South University. Written informed consent to participate in this study was provided by the participants or participants' legal guardian/next of kin.

## Author contributions

YW contributed to the experimental design, performed most of the experiments, prepared the first draft of the manuscript, and contributed to manuscript revision. LX and XXS collected samples. JL, HP, JZ, and YZ performed some of the experiments and analyzed the data. SL, XJS, XL, GH, and ZZ contributed to the study design and provided substantial scientific contribution. ZX proposed the project and contributed to the experimental design and manuscript revision. All authors contributed to the article and approved the submitted version.

## Funding

This work was supported by the National Natural Science Foundation of China (Grant Nos. 81873634 and 82070813), the Hunan Province Natural Science Foundation of China (Grant Nos. 2022JJ30858, 2022JJ30851, 2018JJ2573 and 2020JJ2053), the science and technology innovation Program of Hunan Province (2022RC1010), the Hunan Provincial Innovation Foundation For Postgraduate (Grant No. CX20220120) and the Fundamental Research Funds for the Central Universities of Central South University (Grant No. 2022ZZTS0029).

## Acknowledgments

The m6A immunoprecipitation, microarray hybridization and microarray analysis were performed by Aksomics Company (Aksomics, Shanghai, China).

## Conflict of interest

The authors declare that the research was conducted in the absence of any commercial or financial relationships that could be construed as a potential conflict of interest.

## Publisher’s note

All claims expressed in this article are solely those of the authors and do not necessarily represent those of their affiliated organizations, or those of the publisher, the editors and the reviewers. Any product that may be evaluated in this article, or claim that may be made by its manufacturer, is not guaranteed or endorsed by the publisher.
