# Supplementary figures and images for: The m6A methylation profiles of immune cells in type 1 diabetes mellitus
Source: Front Immunol. 2022 Nov 15;13:1030728. doi: 10.3389/fimmu.2022.1030728 (PMC9707336; doi:10.3389/fimmu.2022.1030728)

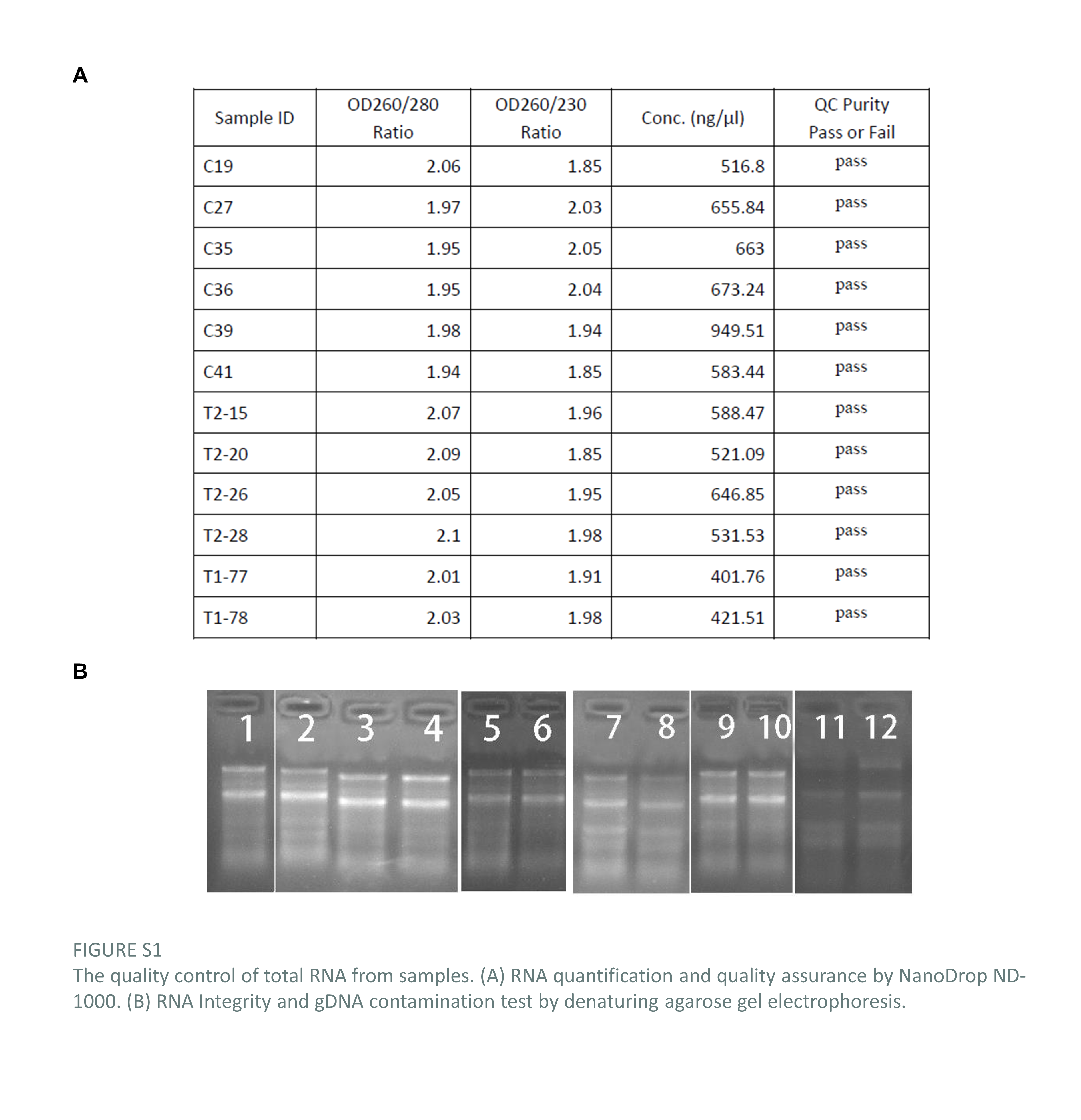

Supplement: Supplementary file 1 [file Image_1.tif]
